# Functional, cognitive and physical outcomes 3 years after minor lacunar or cortical ischaemic stroke

**DOI:** 10.1136/jnnp-2018-319134

**Published:** 2018-12-15

**Authors:** Caroline A McHutchison, Vera Cvoro, Stephen Makin, Francesca M Chappell, Kirsten Shuler, Joanna M Wardlaw

**Affiliations:** 1 Centre for Clinical Brain Sciences, University of Edinburgh, Edinburgh, UK; 2 Centre for Cognitive Ageing and Cognitive Epidemiology, Department of Psychology, University of Edinburgh, Edinburgh, UK; 3 NHS Fife Victoria Hospital, Kirkcaldy, UK; 4 Institute of Cardiovascular and Medical Science, University of Glasgow, Glasgow, UK; 5 UK Dementia Research Institute, University of Edinburgh, Edinburgh, UK; 6 Edinburgh Imaging, University of Edinburgh, Edinburgh, UK

**Keywords:** cognition, walking speed, activities of daily living, dependency, stroke

## Abstract

**Objective:**

Many studies examining stroke outcomes focus on more severe strokes or have short follow-up periods, so the long-term outcomes post-minor ischaemic stroke are unclear.

**Methods:**

We recruited participants from inpatient and outpatient services with a lacunar or minor cortical ischaemic stroke (National Institutes of Health Stroke Scale score <8) and assessed current and premorbid cognitive functioning (Addenbrooke’s Cognitive Examination–Revised (ACE-R), National Adult Reading Test (NART)), physical functioning (Timed Get Up and Go (TUG), 9-Hole Peg Test (9HPT)), dependency (modified Rankin Scale (mRS)), depression (Beck’s Depression Inventory) in-person and remotely (Stroke Impact Scale).

**Results:**

We followed up 224/264 participants at 3 years (mean age at index stroke=67, 126 (56%) men, 25 non-contactable, 15 declined): 66/151 (44%) had cognitive impairment, mean ACE-R 88 (SD 9, range 54–100/100), 61/156 (39%) had depression and 26/223 (12%) were dependent (mRS=3–5). Cognitive impairment at 3 years affected all ACE-R subdomains and was associated with ACE-R 1 year (β=1.054, p<0.001) and NART (β=1.023, p<0.05). Poor physical function was associated with stroke severity (TUG, β=1.064, p<0.01) and recurrent stroke (9HPT, β=1.130, p<0.05 right, β=1.214, p<0.05 left). Higher ACE-R scores were associated with faster TUG (β=−0.279, p<0.05) and 9HPT (right β=−0.257, p<0.05; left β=−0.302, p=0.05) and inversely with dependency (mRS=3–5, OR 0.88, 95% CI 0.80 to 0.97). We adjusted analyses for demographic, stroke and known risk factors. In-person and remote assessments were highly correlated.

**Conclusions:**

Cognitive, physical impairments and depression are common and interrelated 3 years after minor stroke. Cognitive and physical impairments require rehabilitation after minor stroke and argue for better integration of stroke and dementia services.

**Video 1:** 

Stroke is a common cause of disability in adults in high-income countries. Two-thirds of stroke survivors in the UK will have some disability^1^ and a third will be dependent. Stroke is associated with impairments in mobility,^2^ gait and balance^3^ and poor dexterity,^1^ as well as cognitive impairment and dementia.^2 4–6^ These impairments lead to decreased quality of life, low mood and increased mortality, dependency and disability.^4^ Risk factors for post-stroke physical and cognitive impairment overlap and include older age, lower premorbid intelligence (IQ), lower education and more vascular risk factors (eg, atrial fibrillation (AF) and diabetes).^5 7^

Although many studies have documented stroke risk factors and outcomes, most have short follow-up periods and do not focus on minor stroke. Pendlebury and colleagues^5^ found that in 21 hospital-based studies of post-stroke dementia, only 4 (19%) had a follow-up period of 2 or more years, 7 (33.33%) were longitudinal and most included patients with varying severities, mainly moderate strokes.^5 8^ Patients with minor stroke, while less likely to be physically disabled, may have cognitive impairment that restricts their return to full independent function,^9^ but their long-term outcomes are unclear. Furthermore, data on associations between cognitive and physical function, dependency and post-stroke comorbidities like depression are also limited, restricting long-term planning for minor ischaemic stroke.

We aimed to document physical and cognitive impairment and dependency outcomes at 3 years post-minor ischaemic stroke, including any associations between outcomes and specific independent risk factors. We also compared direct in-person with indirect (postal/phone) assessments of several domains.

## Methods

### Participants

In this prospective study, we recruited patients with minor ischaemic stroke (details described previously).^6^ Briefly, we recruited inpatients and outpatients consecutively assessed by the Lothian Stroke Services, Scotland, and diagnosed with minor ischaemic stroke between May 2010 and May 2012 into the Mild Stroke Study 2 (MSS2).

Minor ischaemic stroke was defined as a focal onset of neurological symptoms lasting >24 hours, with no other explanation, a National Institutes of Health Stroke Scale (NIHSS) score of <8 and not expected to result in dependency (modified Rankin Scale (mRS) score <3). Stroke was confirmed by an expert panel based on clinical findings and MRI and subtyped as ‘cortical’ or ‘lacunar’ based on clinical stroke syndrome.^10^

### Follow-up

All participants were invited for follow-up at 1 and 3 years’ post-index stroke. At 3 years, we contacted the general practitioner (GP) to ascertain if the participant was alive before sending a brief questionnaire assessing new diagnoses since the 1-year follow-up. We invited participants to attend a face-to-face assessment by a trained researcher. Where participants could not attend, we offered home visits or telephone interviews. Every effort was made to follow up all participants. Where participants did not respond, we sought further information from the GP and hospital records, and sought cause of death from hospital records and death certificates for participants who were deceased.

### Assessments

The initial follow-up questionnaire collected information on vascular events (eg, stroke, transient ischaemic attack (TIA) and myocardial infarction (MI)), new diagnoses of vascular risk factors and lifestyle factors (eg, smoking and alcohol use) since 1 year of follow-up.

We collected sociodemographic information including socioeconomic status in adulthood (eg, occupation, living situation) and childhood (eg, address, size of house and number of people living in their home at age 11), and education (secondary school or less/further education following secondary school).

We assessed recovery from stroke using the mRS (6=deceased), Clinical Dementia Rating Scale (CDR),^11^ Stroke Impact Scale (SIS)^12^ and European Quality of Life (EQ-5D-5L) scale.^13^ When SIS Emotion Domain responses were inconsistent (eg, *feel sad*=‘none of the time’ and *feel like life is worth living*=‘none of the time’) and the participant could not be contacted for confirmation, responses were removed (n=10).

**Figure 1:**
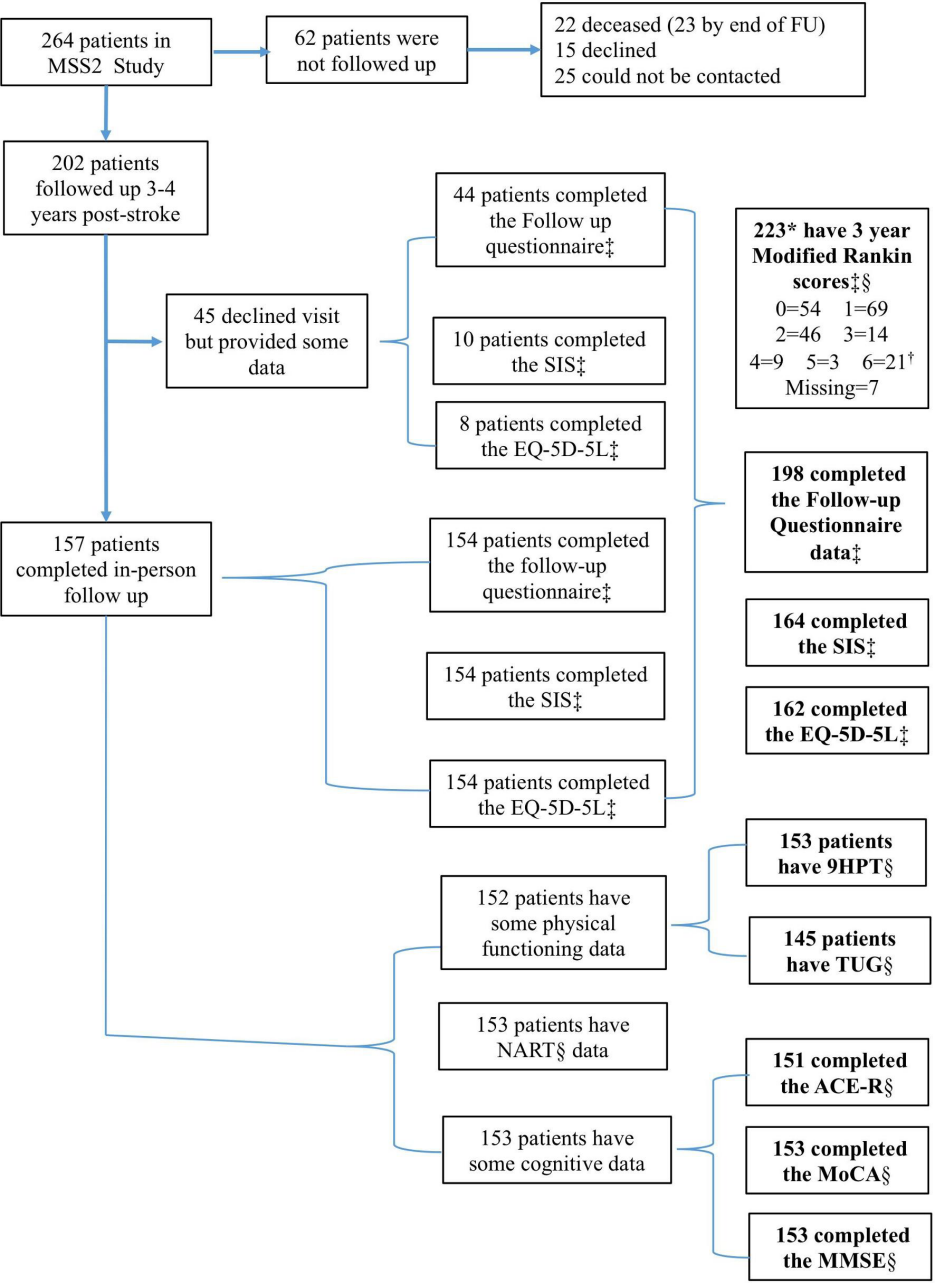
Flow diagram for data collection in the MSS2 3-year follow-up. *Number with self-reported modified Rankin Scale. †Scores of 6 are based on those deceased at 3-year follow-up. ‡Self-reported/subjective measure. §In-person/objective measure. ACE-R, Addenbrooke’s Cognitive Examination–Revised; EQ-5D-5L, European Quality of Life scale; FU, follow-up; MMSE, Mini-Mental State Examination; MoCA, Montreal Cognitive Assessment; MSS2, Mild Stroke Study 2; NART, National Adult Reading Test; 9HPT, Nine Hole Peg Test; SIS, Stroke Impact Scale; TUG, Timed Get Up and Go.

Participants who were not seen for in-person assessment (due to illness or no longer living in the area) were invited to complete the SIS, EQ-5D-5L and sociodemographic questionnaire by post or phone (figure 1 indicates subjective/self-reported and objective/in-person measures).

For those seen in person, we assessed current cognition using the Montreal Cognitive Assessment (MoCA),^14^ Addenbrooke’s Cognitive Examination–Revised (ACE-R)^15^ and Mini-Mental State Examination (MMSE)^16^ and premorbid IQ using the National Adult Reading Test (NART), which provided a standardised estimate of peak cognitive performance in early adulthood.^17^ Where possible, an informant provided information on prestroke changes in cognition using the Informant Questionnaire of Cognitive Decline in the Elderly.^18^ We assessed depression using Beck’s Depression Inventory (BDI).^19^ When participants did not provide a response to question 21 (*Loss of interest in sex*) citing it was not applicable (eg, partner deceased), a score of 0 was assigned (n=12).

We measured physical functioning using the Timed Get Up and Go (TUG),^20^ where participants rise from a chair, walk 3 metres and back returning to a seated position, and the Nine Hole Peg Test (9HPT)^21^ where participants remove nine small pegs one at a time before replacing them. Both tasks are timed.

### Statistical analysis

We analysed cognitive and physical functioning as continuous variables. Where applicable, we categorised scores based on established cut-offs. MoCA scores ≥26 were considered normal.^14^ ACE-R scores were grouped into severe (≤82), mild (83-88) and no cognitive impairment (≥89).^15^ Several cut-offs exist for the MMSE. We considered scores ≥27 as normal.^22^ We used Mann-Whitney U test (U) to compare groups based on levels of cognitive impairment on subscales of the ACE-R and MoCA to determine whether any specific deficit was driving this.

We used linear regression to examine predictors of cognitive and physical functioning and the relationship between these outcomes. Where appropriate, we controlled for known risk factors including demographic (eg, age, sex, further education and occupation), stroke (eg, stroke subtype and stroke severity), vascular risk factors (eg, hypertension, diabetes, hyperlipidaemia and AF) and lifestyle variables (eg, smoking and alcohol consumption). Some outcome variables were log transformed to avoid non-normal data and back transformed for reporting.

We used multinomial logistic regression to examine risk factors for disability (mRS). We grouped participants into no disability (mRS=0), no disability but symptoms present (mRS=1), slight disability (mRS=2), moderate to severe disability (mRS=3–5) and deceased (mRS=6). Based on the most complete available data, we included age at index stroke, sex, stroke type, index stroke severity, recurrent stroke, vascular risk factors and self-reported subjective cognitive difficulties at 1 year (yes/no) as predictors.

## Results

At baseline, 264 patients with clinically confirmed minor ischaemic stroke were recruited.^6^ At 3 years post-stroke, all living participants were followed up and 202/264 (77%) provided data either in person (157, 78%) or by post or phone (45, 22%; figure 1). Those who participated at 3 years (n=202) were more likely to have self-reported subjective cognitive difficulties at 1 year than those without follow-up. There were no significant differences in demographic or index stroke characteristics.

Of the 62 participants without follow-up data, 25 (40%) were not contactable, 15 (24%) declined and 22 (3%) were deceased. Causes of death included cancer (n=13), recurrent stroke (n=3), MI (n=2), heart failure, pulmonary fibrosis, sepsis and unknown (all n=1). As such, outcomes were available for 224 (202 with follow-up data and 22 deceased)/264 (85%) participants, with mean age at index stroke=67, and 126 (56%) men. Of these, 29/223 (13%) experienced a further vascular event including 21 (9%) with recurrent stroke or TIA and 8 (4%) with MI. A new diagnosis of hypertension, hyperlipidaemia, diabetes and/or AF was reported by 67 (30%) participants since the 1-year follow-up (table 1, online supplementary table 1).

10.1136/jnnp-2018-319134.supp1 Supplementary data

**Table 1:** Descriptive statistics at 3-year follow-up

Variable	N	Descriptive
Demographics		
Age at index stroke	224	67.13±11.49
Sex: male n (%)	224	126 (56.25)
Further education: yes n (%)	159	65 (29.15)
Adult SES n (%)	157	
High		49 (31.21)
Middle		49 (31.21)
Low		59 (37.58)
Premorbid IQ	153	112.2±8.44
Stroke characteristics		
Stroke type n (%)	224	
Cortical		131 (58.48)
Lacunar		93 (41.52)
NIHSS worst	224	2.34 (1.32)
3-year outcomes		
Further vascular event: yes n (%)*†	224	
Stroke or TIA		21 (9.37)
MI		8 (3.57)
Disability: mRS† n (%)	217	
No symptoms		54 (24.88)
No significant disability		69 (31.80)
Slight disability		46 (21.20)
Moderate disability		14 (6.45)
Moderate/severe disability		9 (4.15)
Severe disability		3 (1.38)
Dead		22 (10.14)
Cognition‡		
ACE-R	151	88.32±8.92
MoCA	153	25.15±4.03
MMSE	153	27.96±2.48
BDI‡	156	9.81±9.12
Physical functioning‡		
TUG	145	11.91±4.61
9HPT-right	153	28.18±11.68
9HPT-left	153	31.05±15.42

Descriptive statistics are either ±mean and SD or n (%).

*Since 1-year follow-up and including those with further vascular events as cause of death.

†Subjective/self-reported.

‡Objective/in-person assessment.

ACE-R, Addenbrooke's Cognitive Examination - Revised; BDI, Beck's Depression Inventory; 9HPT, Nine Hole Peg Test; MI, Myocardial Infarction; MMSE, Mini Mental State Examination; MoCA, Montreal Cognitive Assessment; NIHSS, National Institutes of Health Stroke Scale; SES, Socioeconomic status; TIA, Transient Ischaemic Attack; TUG, Timed Get Up and Go; mRS, Modified Rankin Scale.

### Cognition

We assessed cognitive functioning in 153/157 (97%) participants seen for in-person assessment. Reasons for no cognitive assessment included visual impairment, English as a second language, declined and not completed (all n=1).

**Figure 2:**
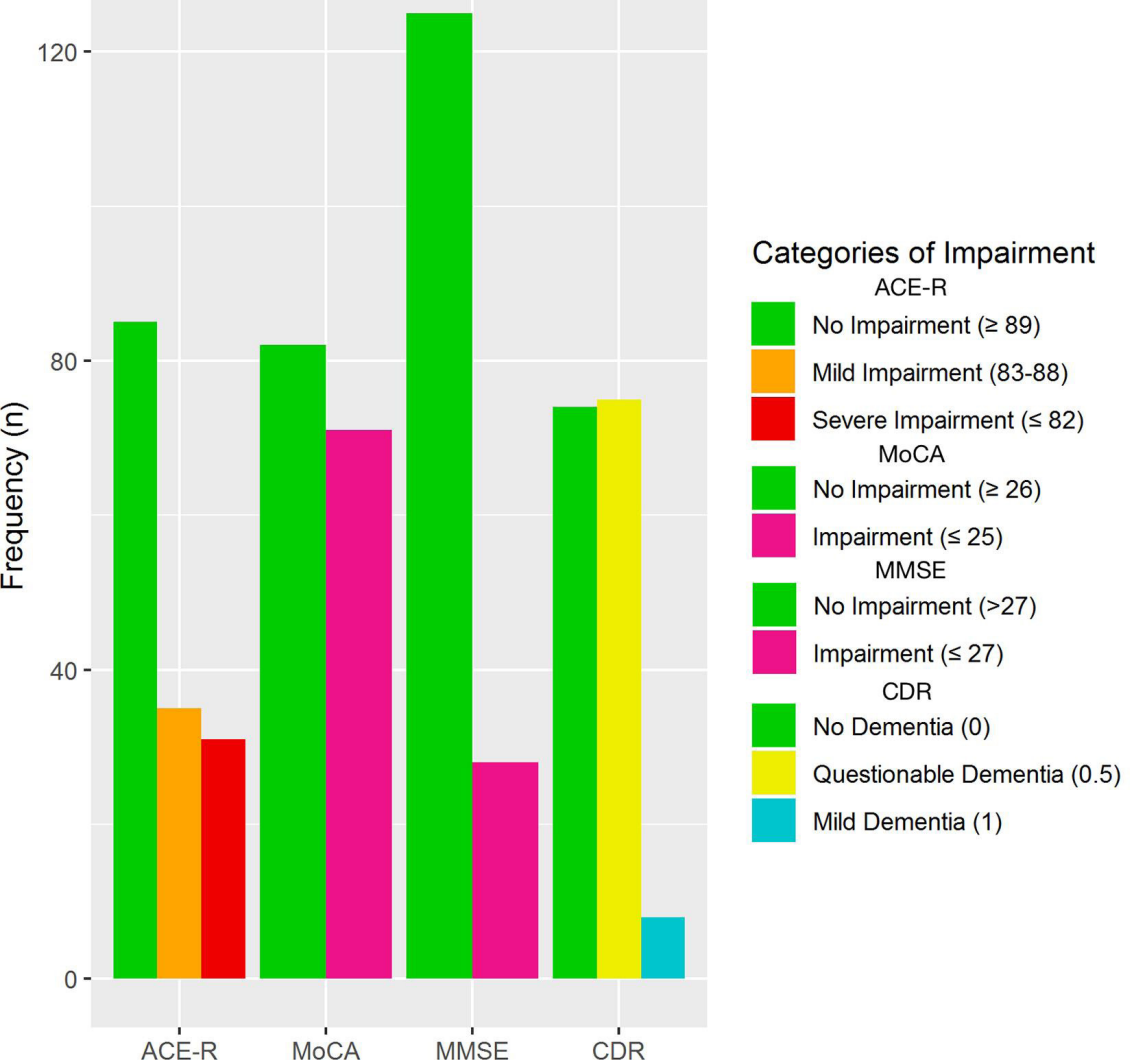
Frequency of cognitive impairment by measure. ACE-R, Addenbrooke’s Cognitive Examination–Revised; CDR, Clinical Dementia Rating Scale; MMSE, Mini-Mental State Examination; MoCA, Montreal Cognitive Assessment.

Scores on the MoCA (n=153) ranged from 10 to 30/30 (M=25.15, SD 4.03) with impairment (MoCA <26) in 71 (46%) participants. Scores on the MMSE (n=153) ranged from 16 to 30/30 (M=27.96, SD 2.48) with impairment in 28 (18.3%) participants. Scores on the ACE-R (n=151) ranged from 54 to 100/100 (M=88.32, SD 8.92) with severe impairment (ACE-R ≤82) in 31 (21%), mild impairment (ACE-R=83–88) in 35 (23%) and normal cognition in 85 (56%) participants (figure 2). Those with impairment showed significantly poorer performance across all subscales compared with those with no impairment on the ACE-R and MoCA (table 2).

**Table 2:** Differences between those with versus without cognitive impairment by subscales

	U†	r
ACE-R		
Attention and orientation	4164*	−0.49
Memory	5218.5*	−0.74
Fluency	5049.5*	−0.69
Language	4493.5*	−0.54
Visuospatial	4159*	−0.44
MoCA		
Orientation	2232*	−0.32
Language	598.5*	−0.70
Executive	1295*	−0.51
Language	1456*	−0.49
Visuospatial	1615*	−0.43

*p<0.001.

†Mann-Whitney U Test.

ACE-R, Addenbrooke’s Cognitive Examination–Revised; MoCA, Montreal Cognitive Assessment.

Scores on the CDR (n=157) ranged from 0 to 1/3. Questionable (CDR=0.5) and mild (CDR=1) symptoms of dementia were reported in 75 (48%) and 8 (5%) participants, respectively (figure 2). No symptoms of dementia (CDR=0) were reported in the remaining 74 (47%).

Age (β=−0.306, 95% CI −0.181 to −0.042), male sex (β=−0.191, 95% CI −2.966 to −0.164), premorbid IQ (NART; β=0.344, 95% CI 0.063 to 0.260), MoCA scores at 1 year (β=0.368, 95% CI 0.188 to 0.639) and hypertension (β=0.328, 95% CI 0.864 to 5.571) predicted MoCA scores at 3 years. Age (B=0.980, p<0.001), ACE-R scores at 1 year (B=1.054, p<0.001) and premorbid IQ (NART; B=1.023, p<0.05) predicted ACE-R scores at 3 years. Sex, further education, adult SES, vascular risk factors and lifestyle factors did not significantly contribute (table 3).

**Table 3:** Predictors of poor cognitive performance at 3-year follow-up

	MoCA		ACE-R	
	β	SE	β	SE
Demographics				
Age at onset	−0.306*	0.035	0.98†	0.006
Sex (male)	−0.191‡	0.704	0.963	0.118
Further education (no)	0.071	0.758	0.834	0.133
Premorbid IQ	0.344*	0.049	1.023‡	0.010
MoCA/ACE-R 1 year	0.368†	0.113	1.054†	0.011
Adult SES				
Middle	−0.140	0.851	1.006	0.147
Low	−0.105	0.922	1.019	0.161
Stroke characteristics				
Stroke subtype (lacunar)	−0.054	0.677	1.065	0.120
Stroke severity—worst (NIHSS)	0.018	0.238	0.968	0.042
Recurrent stroke (yes)	−0.122	0.762	0.912	0.134
Vascular factors				
Diabetes	0.056	0.856	1.028	0.146
Hypertension	0.328*	1.183	1.166	0.200
Hyperlipidaemia	0.098	0.814	0.967	0.140
Atrial fibrillation	0.110	0.779	1.020	0.137
Lifestyle factors				
Smoker				
Previous	−0.004	1.122	1.118	0.198
Current	−1.645	0.735	1.074	0.125
Alcohol (yes)	0.196	1.108	0.999	0.185
Overall model fit (R^2^_adjusted_)	0.490		0.597	

Multivariate analysis controlling for all variables included in the table.

*p<0.01.

†p<0.001.

‡p<0.05.

ACE-R, Addenbrooke’s Cognitive Examination–Revised; MoCA, Montreal Cognitive Assessment; NIHSS, National Institutes of Health Stroke Scale; SES, socioeconomic status.

### Depression

Depression scores (BDI; n=156) ranged from 0 to 49/63 (M=9.81, SD 9.12) with mild depression (BDI=10–18) in 35 (22%), moderate (BDI=19–29) in 61 (39%) and severe (BDI=30–63) in 6 (34%) participants. The remaining 95 (61%) scored within the normal range. Higher BDI scores were associated with male sex (β=0.705, p<0.05) and recurrent stroke (β=1.659, p<0.01). No other demographic, stroke or lifestyle variables significantly contributed.

### Physical outcomes

Physical functioning was assessed in 153/157 (97%) participants seen for in-person assessment. Four did not complete the 9HPT (poor vision (n=2), ran out of time and did not consent (both n=1)) and 12 did not complete the TUG (mobility difficulties (n=6), did not consent or declined (n=4), ran out of time and other (both n=1)).

Scores on the TUG (n=145) ranged from 3 to 39 s (M=12, SD 5). Scores on the 9HPT (n=153) ranged from 14 to 110 s (mean=28, SD 12) and 16–149 s (M=31, SD 15) for right and left hand, respectively. Based on normative data for patients with acute stroke 6 months post-stroke,^23^ four (3%) participants were impaired on their right and seven (5%) on their left hand. Poorer TUG performance was associated with slower right-handed (β=0.086, p<0.05) and left-handed 9HPT times (β=0.201, p<0.05) when controlling for age, stroke severity and recurrent stroke.

When controlling for demographic, stroke characteristics, vascular factors and lifestyle variables, age and recurrent stroke predicted slower 9HPT right (β=1.011, p<0.001 and β=1.30, p<0.05, respectively) and left hand performance (β=1.012, p<0.001 and β=1.214, p<0.05, respectively). Slower TUG times were associated with age (β=1.009, p<0.01) and stroke severity (β=1.064, p<0.01; table 4).

**Table 4:** Predictors of poor physical functioning and higher depression scores at 3-year follow-up

	TUG		9HPT—right hand		9HPT—left hand		BDI	
	β	SE	β	SE	β	SE	β	SE
Demographics								
Age at onset	1.009*	0.003	1.011†	0.002	1.012†	0.003	1.009	0.008
Sex (male)	1.024	0.059	1.079	0.049	1.108	0.052	0.705‡	0.153
Further education (no)	0.970	0.066	0.968	0.053	0.985	0.058	0.998	0.168
Adult SES								
Middle	1.072	0.074	1.028	0.061	1.014	0.065	0.994	0.191
Low	1.049	0.075	1.059	0.062	0.968	0.066	1.228	0.192
Stroke characteristics								
Stroke subtype (lacunar)	1.052	0.061	1.062	0.051	1.039	0.054	0.866	0.156
Stroke severity—worst (NIHSS)	1.064*	0.023	1.023	0.018	1.011	0.019	1.068	0.055
Recurrent stroke (yes)	1.023	0.067	1.130‡	0.055	1.214‡	0.058	1.659*	0.169
Vascular factors								
Diabetes	1.103	0.076	0.917	0.063	1.049	0.067	0.945	0.195
Hypertension	0.924	0.101	1.003	0.086	0.871	0.091	0.865	0.267
Hyperlipidaemia	0.924	0.073	0.941	0.059	0.882‡	0.063	0.966	0.183
Atrial fibrillation	1.002	0.071	1.005	0.059	1.009	0.063	0.981	0.182
Lifestyle factors								
Smoker								
Previous	1.030	0.089	1.013	0.074	1.056	0.080	1.358	0.234
Current	0.973	0.067	1.048	0.056	1.044	0.060	1.336	0.174
Alcohol (yes)	0.959	0.086	0.922	0.069	0.891	0.075	0.620	0.213
Overall model fit (R^2^_adjusted_)	0.044		0.148		0.195		0.140	

Multivariate analysis controlling for all variables included in the table.

*p<0.01,

†p<0.001.

‡p<0.05.

BDI, Beck's Depression Inventory; 9HPT, Nine Hole Peg Test; NIHSS, National Institutes of Health Stroke Scale; SES, socioeconomic status; TUG, Timed Get Up and Go.

### Physical, cognitive and functional outcomes

When controlling for age, higher ACE-R scores were associated with faster TUG (β=−0.279, p<0.05) and 9HPT times (right hand: β=−0.257, p<0.05; left hand: β=−0.302, p=0.05). Lower ACE-R scores were associated with increased risk of moderate to severe disability (mRS=3–5; OR 0.88, 95% CI 0.80 to 0.97) when controlling for age and premorbid IQ.

### Disability and recovery after stroke

Disability was measured using self-reported (n=217, including those who were deceased) and interviewer-reported mRS (n=156). Moderate to severe disability (mRS=3–5) was reported in 26/217 (12%) and 19/156 (12%) participants in self-reported and in-person assessment, respectively (table 1).

Moderate to severe disability (mRS=3–5) was associated with age (OR 1.06, 95% CI 1.01 to 1.12), stroke severity (OR 2.29, 95% CI 1.48 to 3.36), recurrent stroke (OR 5.25, 95% CI 1.63 to 20.48) and self-reported cognitive difficulties at 1 year (OR 6.53, 95% CI 2.11 to 20.22). Death (mRS=6) was associated with age (OR 1.10, 95% CI 1.05 to 1.18). Sex, stroke type and vascular risk factors were not associated with disability. Self-reported cognitive difficulties at 1 year were also associated with increased risk of symptoms without disability (mRS=1; OR 3.00, 95% CI 1.31 to 6.82) and slight disability (mRS=2; OR 5.10, 95% CI 2.05 to 12.68).

### In-person versus indirect assessments

We used the SIS (n=166) and EQ-5D-5L (n=163) to measure self-reported post-stroke recovery with higher scores indicating better functioning. Overall, self-reported functioning was good with median scores of 1/5 across the EQ-5D-5L domains and average scores on the SIS domains ranging from 82.27/100 (Strength Domain) to 90.34/100 (Communication Domain; online supplementary table 1).

Higher ACE-R scores (β=0.41, bootstrapped (2000 replications) 95% CI 0.33 to 1.32) were associated with better SIS Memory domain scores when controlling for age and premorbid IQ. Faster TUG performance was associated with better SIS Mobility domain scores (β=−0.63, 95% CI −2.87 to −1.86) and faster right and left 9HPT performance was associated with better SIS Hand Function domain scores (β=−0.40, bootstrapped (2000 replications) 95% CI −1.06 to −0.46 and β=−0.48, bootstrapped (2000 replications) 95% CI −0.91 to −0.46, respectively) when controlling for age. Lower SIS Emotion domain scores were associated with higher BDI scores (β=−0.77, 95% CI −1.80 to −1.37).

## Discussion

This prospective, long-term study of outcomes at 3 years post-minor ischaemic stroke, at an average age of 67, identified impairments in cognitive functioning in almost half (47%) of the 153/202 patients available for the assessment, with around a third being impaired in physical functioning, 13% having mRS score indicating dependency and 29/224 (13%) having a further TIA/minor stroke or MI between 1 and 3 years of follow-up. Cognitive dysfunction affected all subdomains of the ACE-R, was consistent across all measures of current cognition, and was associated with higher levels of physical dysfunction and disability. Lower premorbid IQ, poorer cognition at 1 year and older age predicted cognitive dysfunction, while index stroke severity and recurrent stroke predicted physical dysfunction. Data on the long-term outcomes post-minor ischaemic stroke are sparse since most studies report outcomes at 3 to 6 months and include more severe strokes. We show that important long-term negative consequences are common after minor stroke, particularly in cognition, even in a relatively young population with potentially >20 years’ life expectancy.

### Cognition

Our findings using the ACE-R support previous research in (mostly) patients with acute stroke with a range of stroke severities at 1–5 years of follow-up (n=55)^24 25^ and reflect the global nature of this dysfunction found using full neuropsychological testing.^26^ Fluctuations in cognition shortly following stroke are common,^26^ but long-term improvements, particularly at 1 year, have been shown.^26 27^ As follow-up past 1 year is uncommon, it is not clear whether these improvements are temporary. Some evidence suggests that the risk of cognitive impairment increases for up to 5 years at a rate of 3% per year.^5^ However, these findings are not specific to patients with minor stroke. Pre-stroke cognitive decline is associated with post-stroke cognitive decline.^5 28^ However, pre-stroke cognitive decline differs from premorbid IQ, the latter representing an individual’s peak cognitive ability in early adulthood prior to any neurological damage or ageing effects. Premorbid or childhood IQ correlates strongly with adulthood intelligence,^29^ risk of cognitive impairment and dementia, particularly vascular dementia.^30^ Our findings show that lower premorbid IQ, but not necessarily pre-stroke cognitive decline, is associated with higher risk of poorer cognition post-stroke.

The association between childhood/premorbid IQ and education has been widely discussed and many use education as a proxy for IQ given its close relationship.^31^ However, the relationship between these variables is complex and few studies have examined their independent association with health outcomes, particularly with stroke. We show that premorbid IQ (as measured by NART) associated with post-stroke cognition, independent of the education. At the 1-year follow-up, both NART and years of education were significant independent predictors^32^ suggesting that these variables should be treated separately. However, our education variable may lack statistical power; therefore, further research is needed to determine the independent association of these variables with stroke risk and outcomes.

Although previous research has shown significant associations between stroke severity and cognition,^5^ we do not find these associations to be significant in our cohort. This is likely due to the small range in stroke severities included in this study.

### Physical functioning

Physical functioning varied greatly, particularly on the 9HPT. Although no published norms for patients with stroke on the TUG exist, several studies including patients with stroke of a similar age used a cut-off of 12 s.^33^ Based on this, 46 (32%) participants in the MSS2 would be classified as impaired. Furthermore, 37% of participants reported some degree of disability (mRS ≥2) indicating loss of independence and difficulties in activities of daily living (ADL).

Although hand dysfunction is common in patients with stroke,^34 35^ comparison of 9HPT scores with normative data for patients 6 months post-stroke showed that impairments in dexterity were uncommon in the MSS2.^23^ These findings were supported by generally high SIS Hand Function scores. We were unable to identify any studies which examined dexterity, gait and walking speed in patients with minor stroke after more than 6 months, so it is possible that hand functioning continues to improve; the association between poorer 9HPT and recurrent stroke in the MSS2 supports the suggestion that hand functioning is sensitive to the impact of stroke.

### Strengths and limitations

We followed up 224/264 participants (85%) at 3 years post-stroke. Most were seen in-person, but where this was not possible, questionnaire follow-up was conducted by phone or post to maximise response rate. We found no differences between those with and without follow-up. Our follow-up was comprehensive, assessing physical and cognitive functioning, subjectively and objectively, and causes of death. This allowed us to determine the rates of difficulties experienced in daily life as well as the difficulties that would be seen by clinicians. The large sample allowed us to adjust for potential confounders and test for independent risk factors relevant to clinical practice and patients.

We used cognitive screening tools sensitive to detecting impairment in patients with stroke^24^ and assessed premorbid IQ. Cognitive screening tools are quick and easy to administer but may lack sensitivity to impairments in other areas of cognition (eg, processing speed and areas of executive functioning). Detailed neuropsychological testing would be needed to determine the full effects of minor stroke; however, this would increase assessment time, participant burden, fatigue and risk of missing data.

We defined minor ischaemic stroke using an NIHSS score <8; however, many definitions exist.^36^ An NIHSS score ≤3 has been associated with favourable short-term and long-term outcomes and has been suggested as a reliable definition.^36^ In our sample, 202/264 (77%) met this criteria at baseline (158/202, 78% at 3 years’ follow-up). Participants with follow-up and cognitive and physical assessment did not differ on the initial NIHSS compared with those without. We controlled for stroke severity in our analyses; therefore, it is unlikely that our findings are influenced by those with more severe strokes.

Individuals unable to undergo MRI at baseline were excluded, which may have introduced bias in our sample. However, this is likely to be minimal as we include patients with minor stroke only and contraindications to MRI were not related to index stroke characteristics.

### Implications

Physical dysfunction is frequently the focus in post-stroke clinical practice as difficulties are more obvious as stroke severity increases. We show that those with minor stroke, hence less obvious physical dysfunction, experience high levels of disability and difficulties in ADL. Furthermore, identification of those with physical dysfunction, via risk factors such as stroke severity and recurrent stroke, may help identify those with cognitive impairment, even when the impairment is mild.

Importantly, cognitive dysfunction, though common, could easily go unnoticed without objective assessment. Even when mild, cognitive dysfunction can have a substantial impact on the patient’s independence and ability to monitor and control other risk factors (eg, take antihypertensive medications, maintain a healthy diet) and thus maintain brain health.^37^ These findings highlight the multidimensional effects of stroke and the possible benefits of considering premorbid IQ when determining risk of post-stroke cognitive dysfunction.

## Summary

Studies examining the long-term cognitive and physical outcomes following minor ischaemic stroke are sparse. Our findings show long-term cognitive and physical dysfunction is common and that cognition may further negatively impact on their physical function and on their ADL. More research is required to identify the changes in functioning over the course of recovery post-minor stroke to help better understand their trajectories.
