# Supplementary material for: Functional, cognitive and physical outcomes 3 years after minor lacunar or cortical ischaemic stroke
Source: J Neurol Neurosurg Psychiatry. 2018 Dec 15;90(4):436–43. doi: 10.1136/jnnp-2018-319134 (PMC6581154; doi:10.1136/jnnp-2018-319134)
Supplement: Supplementary data [file jnnp-2018-319134supp001.pdf]

Supplemental Data

Table 1. Frequencies at 3year follow-up

Variable	N	Descriptive
**Vascular outcomes**		
Vascular risk factors		
Hypertension	198	12(6.06)
Hyperlipidaemia	197	52 (26.40)
Diabetes	195	15 (7.69)
Atrial fibrillation	195	22 (11.28)
**Disability**		
mRS[a] n(%)	156	
No Symptoms		61 (39.10)
No significant disability		59 (37.82)
Slight disability		17 (10.90)
Moderate disability		18 (11.54)
Moderate/severe disability		1 (0.64)
Severe disability		0
**CDR[a]**	157	
No dementia		74 (47.13)
Very mild dementia		75 (47.77)
Mild dementia		8 (5.10)
**SIS[bc]**		
Strength	165	82.27±22.15
Memory	164	82.58±18.17
Emotion	155	79.91±19.40
Communication	165	90.34±13.92
ADL	165	86.98±18.72
Mobility	165	83.77±20.77
Hand function	166	84.34±23.83
Social	166	83.30±24.10
**EQ-5[bd]**		
Mobility	162	1 (1-3)
Self-care	163	1 (1-1)
Usual activities	163	1 (1-2)
Pain/discomfort	163	1 (1-2)
Anxiety/depression	163	1 (1-2)

Descriptive statistics are either ± Mean and SD, n(%) or median (IQR).

[a] Objective/In-person assessment
[b] Subjective/Self-reported
[c] Possible scores ranged from 1-100 which higher scores indicating better functioning.
[d] Possible scores ranged from 1-5 which higher scores indicating better functioning.

mRS = Modified Rank Scale; CDR = Clinical Dementia Rating; SIS = Stroke Impact Scale; ADL = Activities of Daily Living; EQ-5 = European Quality of Life Scale
